# Surface-Water Nitrate Exposure to World Populations Has Expanded and Intensified during 1970–2010

**DOI:** 10.1021/acs.est.3c06150

**Published:** 2023-11-21

**Authors:** Junjie Wang, Xiaochen Liu, Arthur H. W. Beusen, Jack J. Middelburg

**Affiliations:** † Department of Earth Sciences, Utrecht University, Utrecht 3584CB, The Netherlands; ‡ PBL Netherlands Environmental Assessment Agency, The Hague 2500GH, The Netherlands

**Keywords:** nitrate exposure, human health risks, surface waters, global assessment, multiscale distributions, long-term changes

## Abstract

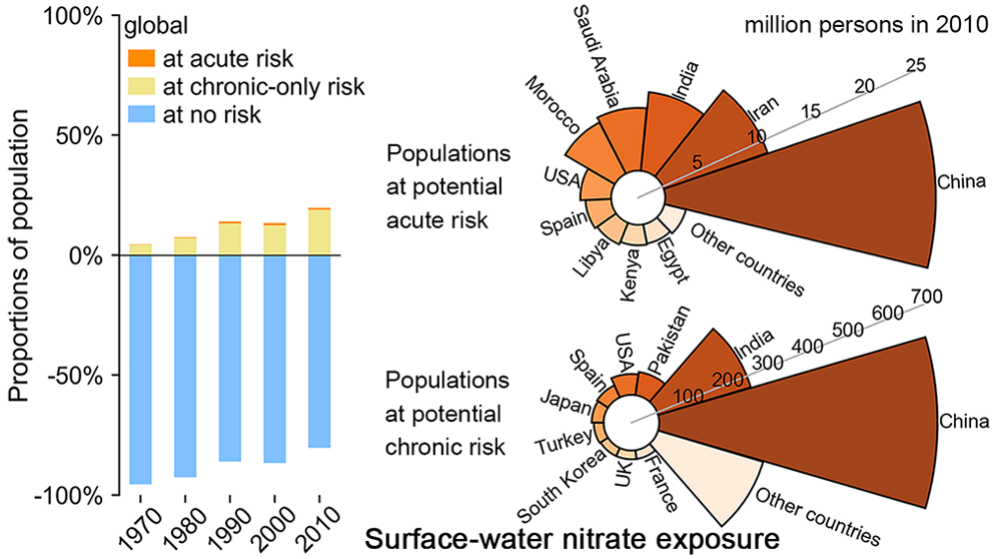

Excessive nitrate in surface waters deteriorates the water quality and threatens human health. Human activities have caused increased nitrate concentrations in global surface waters over the past 50 years. An assessment of the long-term trajectory of surface-water nitrate exposure to world populations and the associated potential health risks is imperative but lacking. Here, we used global spatially explicit data on surface-water nitrate concentrations and population density, in combination with thresholds for health risks from epidemiological studies, to quantify the long-term changes in surface-water nitrate exposure to world populations at multiple spatial scales. During 1970–2010, global populations potentially affected by acute health risks associated with surface-water nitrate exposure increased from 6 to 60 million persons per year, while populations at potential chronic health risks increased from 169 to 1361 million persons per year. Potential acute risks have increasingly affected Asian countries. Populations potentially affected by chronic risks shifted from dominance by high-income countries (in Europe and North America) to middle-income countries (in Asia and Africa). To mitigate adverse health effects associated with surface-water nitrate exposure, anthropogenic nitrogen inputs to natural environments should be drastically reduced. International and national standards of maximum nitrate contamination may need to be lowered.

## Abstract 2

This paper presents a global assessment of populations at potential acute and chronic health risks associated with surface-water nitrate exposure and their changes during 1970−2010.

## 1 Introduction

Water-related health risks are one of the major concerns worldwide. Today, more than two billion, or one in four people, around the world do not have safe drinking water services.^1^ The quality of the drinking-water supply is critical to human health, particularly in poor regions with water scarcity and/or limited capabilities of implementing costly treatments to improve water quality for drinking.^1−3^ Unsafe water sources are responsible for 1.4 million global deaths per year^4^ and 5% of deaths in low-income countries.^5^

Surface waters provide half of the current global drinking water supply.^6^ Despite increasing efforts in drinking-water management over the past two decades, in 2022, more than one million people still directly drank surface waters without any treatments.^1^ The quality of surface waters may thus pose vital influences on human health, as drinking water is one of the most important pathways of human exposure to potential adverse chemicals.

Nitrate is a reactive form of dissolved nitrogen that supports primary productivity and is ubiquitous in natural environments. In humans, nitrate can be generated in vivo, and this endogenous nitrate production may benefit human health due to its antibacterial role in protecting the gastrointestinal tract against a variety of pathogens.^7^ External nitrate intake through drinking water and diet is the major source of human nitrate exposure,^8^ which, if high, may trigger adverse health effects.^9^ Compared with populations drinking nitrate-free water, populations drinking water with 47 mg/L of nitrate ion (unified unit used in this paper, hereafter referred to as mg/L) have a more than doubled daily nitrate intake, and those drinking water with 84 mg/L of nitrate have a nearly tripled daily nitrate intake.^10^ At high levels of nitrate contamination (50 mg/L or higher), drinking water contributes 70% or more of total daily nitrate intake.^11^

Various adverse health effects have been reported in relation to human exposure to high levels of nitrates in the water supply. Methemoglobinemia is an acute adverse health effect of high nitrate exposure, particularly for infants, pregnant women, and individuals with gastrointestinal or genetic diseases.^7,12−14^ Under high waterborne nitrate exposure, elevated nitrate intake increases endogenous nitrite formation via reduction of nitrate under anaerobic conditions in the digestive tract. This, in turn, enhances the binding of nitrite with hemoglobin in red blood cells to form methemoglobin and reduces the hemoglobin’s oxygen-carrying capacity.^7^ Infants tend to be more susceptible to methemoglobinemia because of their larger fluid intake (e.g., water and/or formula made with water containing high levels of nitrate) relative to their body weight, their less acidic gastrointestinal system (which enhances invasion of bacteria and reduction of nitrate to nitrite), their larger proportion of readily oxidizable fetal hemoglobin, their lower methemoglobin reductase activity (an enzyme that converts methemoglobin back to hemoglobin), and their higher susceptibility to gastroenteritis than adults.^8,15,16^ Symptoms of methemoglobinemia include cyanosis, headache, stupor, fatigue, tachycardia, coma, convulsions, and asphyxia.^17^ Methemoglobinemia is life-threatening when methemoglobin levels exceed 10%.^13,18^ The World Health Organization (WHO)’s nitrate guideline of 50 mg/L^7^ and the national maximum contaminant level of 45 mg/L in many countries^19−23^ for nitrates in water supplies are set to protect against methemoglobinemia.

Epidemiological studies have also reported various chronic health effects potentially related to exposure to high nitrate from drinking water. Nitrate is a precursor in the in vivo formation of *N*-nitroso compounds during endogenous nitrosation, and most of the *N*-nitroso compounds are carcinogenic and teratogenic. High nitrate ingestion may therefore increase the risks for various cancers, birth defects, and spontaneous abortions.^24^ Documented adverse health risks range from thyroid hypertrophy,^7,25−27^ non-Hodgkin’s lymphoma,^28,29^ insulin-dependent diabetes mellitus,^30^ central nervous system birth defects,^31^ and intrauterine growth restriction^32^ to cancers of the digestive tract (e.g., stomach, esophagus, colon, and rectum)^29,33−39^ and the genitourinary system (e.g., bladder, ovarian, prostate).^34,39−42^ Moreover, associations with high nitrate exposure tend to be stronger for aggressive forms of cancer than for the less aggressive form.^40,41^ Although these health problems are complicated by other causes from genetic diseases to endogenous disorders, and chemical or drug exposure, high nitrate exposure may play a role therein as a cofactor and adds to the exposome and associated health risks. More importantly, these chronic risks have been reported to occur with ingested nitrate exposure well below the WHO’s guideline (50 mg/L) and many countries’ maximum contaminant level (45 mg/L).^7,19−23^

Over the past 50 years, surface-water nitrate contamination has been rising in developed and developing regions around the world due to intensified human activities, such as intensive agriculture, nitrogen-based fertilizer use, livestock farming, land-use change, population growth and centralization, industrialization, and wastewater discharge^43,44^ (Figure S1). In many surface-water systems worldwide, nitrate has been the dominant form of nitrogen and its concentrations have been observed to increase over the past decades.^45−49^ The increased surface-water nitrate concentrations have become a pressing environmental concern because they cause eutrophication and associated harmful algal blooms, hypoxia, and fish deaths, posing detrimental impacts on water quality and ecosystem functioning.^15,50^ Moreover, they may affect the availability and quality of drinking water and, in turn, trigger the aforementioned potential health risks to humans. However, it remains unclear how potential health risks associated with the changing surface-water nitrate exposure and their affected populations have changed worldwide over the past 50 years.

Surface-water nitrate exposure is an emerging health issue that not only changes with time but also differs across regions. Dictated by hydroclimatic conditions and socioeconomic development levels, surface-water nitrate exposure would be higher and trigger more symptomatic health risks to inhabitants in areas with high and increased anthropogenic nitrogen loading, large water stress, limited water treatment capabilities, and poor accessibility to safely managed drinking water.

Health risks related to high nitrate exposure are preventable if nitrate exposure and its affected populations are identified and quantified and if focused controlling action in its hotspot regions can be taken. Assessing the long-term changes and regionalization in global surface-water nitrate exposure and its potential effects on human health is an essential premise of managing health risks related to water pollution toward achieving the United Nations Sustainable Development Goals (SDGs)^51−53^ of human well-being, water security, and associated inequality reduction. Such a global-scale assessment is instrumental in motivating the understanding of the exposome and the role of the environment in human diseases.

This study aims to address this knowledge gap by quantifying the spatial and temporal changes in surface-water nitrate exposure to world populations and their implications for potential acute and chronic health risks. We used the spatially explicit surface-water nitrate concentrations simulated by the Integrated Model to Assess the Global Environment (IMAGE)^54^-Dynamic Global Nutrient Model (DGNM)^55−57^ and gridded population density data from the same integrated model IMAGE^54^ with a consistent resolution of 0.5° × 0.5°. In combination with thresholds for various health risks from epidemiological studies, we calculated world populations at potential acute and chronic health risks related to surface-water nitrate exposure at multiple spatial scales during 1970–2010. The assessment in this work is motivated by the recognition of intensive anthropogenic impacts on the fate of nitrate in the global nitrogen cycle and its potential effects on human health. To the best of our knowledge, this work is the first global-scale assessment of surface-water nitrate exposure and associated health risks in a changing world. This novel assessment leverages biogeochemical, socioeconomic, and epidemiological knowledge from different disciplines, and evaluates the role of humans in driving environmental changes and environmental effects on humans in a consistent manner.

## 2 Materials and Methods

### 2.1 Global Nutrient Model and Surface-Water Nitrate

Measurement data of nitrate concentrations do not cover all locations in global surface waters in the long historical span from 1970 to 2010. The use of a global biogeochemical nutrient model can fill the gap in the spatial and temporal coverage of measurement data.

In this study, we used the global spatially explicit, mechanistic, integrated, dynamic nutrient model IMAGE-DGNM,^55,56^ which can reproduce the historical global surface-water nitrate concentrations at a 0.5° × 0.5° resolution per year during 1970–2010 (Figure S2). Within the IMAGE-DGNM framework, the IMAGE model^54^ provides the long-term changes in the spatial distributions of land cover, population, climate, and water use, the hydrology model PCR-GLOBWB^58,59^ simulates the changes in surface-water area, volume, runoff, and discharge in different waterbodies (e.g., rivers, lakes, and reservoirs) for each year, and the DISC module^55^ resolves the coupled dynamic in-stream biogeochemical processes in surface waters. The coupled model tracks reactive nitrogen forms, including nitrate from various natural and anthropogenic sources and processes on land, their transformations and transport across inland-water networks, and their changed concentrations spatially and temporally. Detailed model descriptions of IMAGE-DGNM can be found in Beusen et al.,^60^ Vilmin et al.,^55^ Wang et al.,^56^ and Wang et al.^57^ Extensive validations of the IMAGE-DGNM simulations in global surface waters have been performed in previous publications.^55−57,60−63^ In this study, we further validated the IMAGE-DGNM simulated surface-water nitrate concentrations against long-term site-level observational data for a range of major river basins worldwide since the 1950s from the literature and databases, including USGS,^64^ GEMStat,^65^ and GLORICH^66^ (Table S1). These sites and surface-water systems cover different continents, climate zones, hydrological settings, and human activities (e.g., economic development levels, agriculture production, population, and land use). We compared simulations and observations site-to-site year-by-year, examined their long-term trends, and calculated their root mean square error (RMSE) values for further statistical evaluation (Figure S3). The comparisons show that the simulated surface-water nitrate concentrations generally agree with observations at different sites and surface-water systems since the 1950s (Figure S3). The RMSE values for the validation sites range from 25% to 83%, and their long-term historical trends have been reasonably reproduced in the simulations (Figure S3).

It is important to recognize that in regions and countries with managed drinking-water services, water utilities may adopt different treatments of surface water, and some may include decreasing nitrate to make it safer for drinking, such as diluting the contaminated water with a low-nitrate source, ion exchange, biological denitrification, reverse osmosis, and electrodialysis.^7^ However, these nitrate treatment processes have disadvantages of high costs, operational complexities, and the need for disposal of resin, brine, or reject water,^7^ and are rarely included in common municipal water treatment processes. Conventional municipal water treatment processes are not effective for nitrate removal because of the stability of nitrate ion against coprecipitation and adsorption. In addition, ammonia from surface waters and/or added for chloramination during the municipal water treatment can, as a substrate, enhance nitrification and increase the nitrate in the distribution systems.^67^ Moreover, spatial and temporal data of national or regional surface-water treatment technology and capacity are not available. Given the limited adoption of nitrate removal process(es) in the common municipal water treatment, the aforementioned uncertainty related to nitrate produced from ammonia in the distribution systems, and the unavailability of national/regional treatment data, we assume that the use of surface-water nitrate concentrations is reasonable to study surface-water nitrate exposure and associated health risks.

### 2.2 Gridded Population Density Data

The annual gridded population density data with a 0.5° × 0.5° resolution for the period 1970–2010 were obtained from the published data sets of the same integrated model IMAGE^54^ (https://www.pbl.nl/en/image/data). In the IMAGE model, the yearly national-scale population estimates from United Nations World Population Prospects^68^ were downscaled to a 0.5° × 0.5° resolution based on the approach of Van Vuuren et al.^69^ In the integrated model, population and its spatiotemporal changes act as important drivers of socioeconomic changes and have further consequences in the Earth system, including water and air pollution, climate change, and biodiversity loss.^54^ By utilizing the same, internally consistent set of data sources and modeling approaches for both population and the driven surface-water nitrate concentrations, it becomes feasible to track the role of humans in driving environmental changes while also assessing the consequent environmental effects on humans in a consistent manner. In order to ensure the consistency of the temporal coverages of the data of all model components, we focused on addressing the long-term change from 1970 until 2010, and 2010 is the latest year for which the data of all model components are available.^54,56^

### 2.3 Potential Health Risk Levels Associated with Surface-Water Nitrate Exposure

To quantify the spatial distributions and temporal changes of the potential health risks related to surface-water nitrate exposure, we compiled the literature-reported health risks associated with high waterborne nitrate exposure in Table S2. We also summarized the threshold nitrate concentrations above which associated health risks were reported to increase. These risks range from some acute diseases (methemoglobinemia) to chronic adverse effects (e.g., carcinogenicity). According to Table S2, the threshold nitrate concentration for potential acute risk is set at 50 mg/L of nitrate ions, in line with the WHO’s guideline.^7^ To determine the threshold nitrate concentration for analyzing potential chronic risks, we took the lower boundaries of the threshold ranges for the six types of chronic risks reported in epidemiological studies (Table S2) and calculated their average. This average (11 mg/L) is thus considered as the threshold nitrate concentration for potential chronic risks.

When surface-water nitrate concentrations exceed a threshold, local inhabitants are assumed to be at potential acute or chronic health risks associated with surface-water nitrate exposure correspondingly. Otherwise, local inhabitants are assumed to have no potential acute or chronic health risk associated with surface-water nitrate exposure. The potential acute or chronic health risk level (PRL_*i*_) associated with surface-water nitrate exposure at grid *i* for an analyzing year can be calculated as
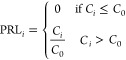
1where *C*_0_ is the threshold nitrate concentration for potential acute or chronic risks and *C*_*i*_ is the nitrate concentration at grid *i* for the analyzing year.

Therefore, when PRL_*i*_ is larger than 0, populations at grid *i* are assumed to be exposed to potential acute or chronic health risks associated with surface-water exposure for the analyzing year. We discussed exposed populations and their temporal changes across multiple scales, ranging from national,^70^ to continental and global levels. We specifically examined the relative percentages of changes in the affected population across various scales, which may be more illustrative than absolute population numbers in discerning the trends, locations, and magnitudes of evolution in surface-water exposure and associated health effects.

## 3 Results

### 3.1 Global Populations at Potential Risks Associated with Surface-Water Nitrate Exposure

**Figure 1:**
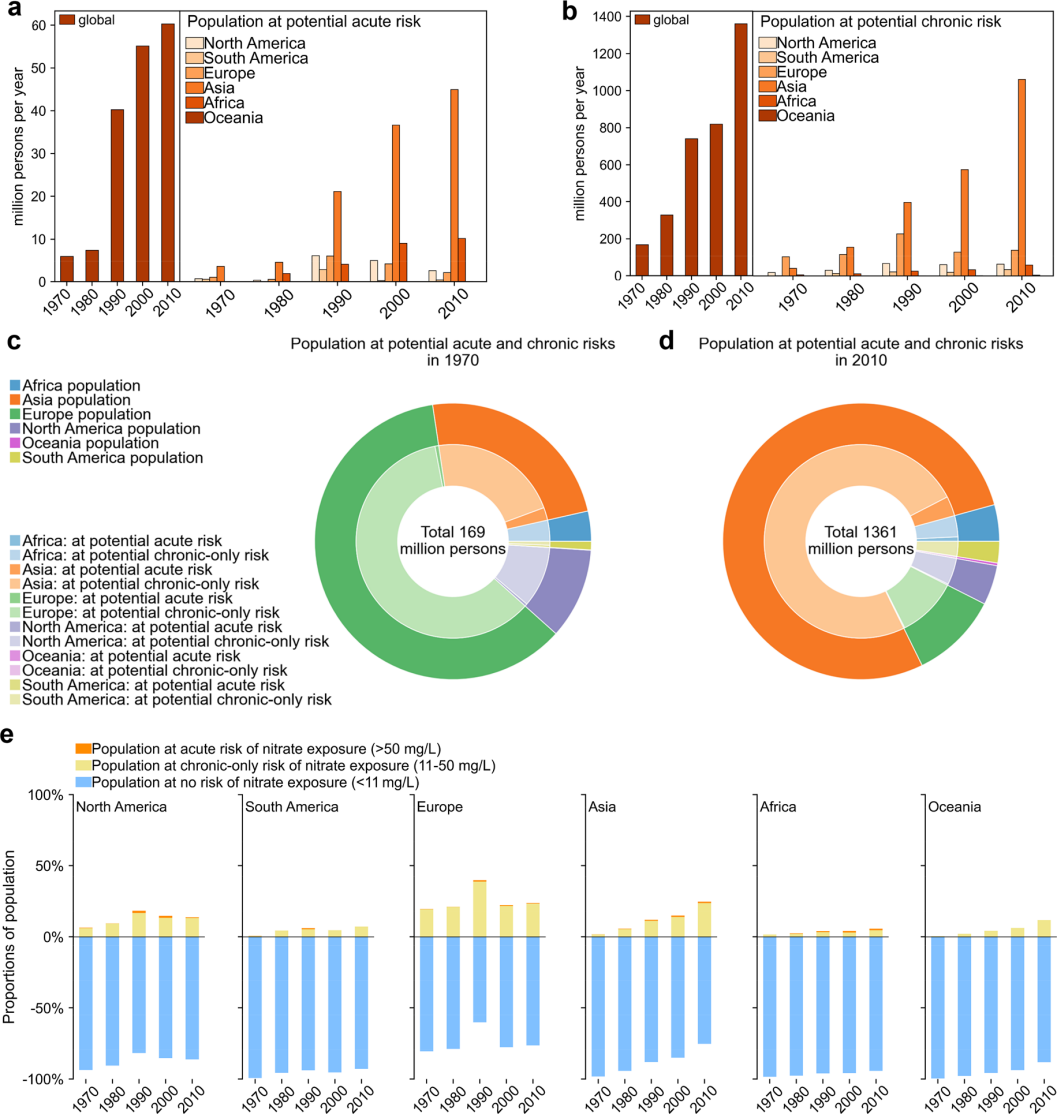
Temporal changes in global continental populations at potential acute (a) and chronic (b) risks associated with surface-water nitrate exposure during 1970–2010, contributions of different continents to world populations at potential acute and chronic-only risks associated with surface-water nitrate exposure in 1970 (c) and 2010 (d), and temporal changes in continental proportions of populations at potential no risk, chronic-only risk, and acute risk associated with surface-water nitrate exposure (e). In (c–e), populations at chronic-only risk are populations at chronic risk excluding those at acute risk.

During 1970–2010, global populations potentially affected by acute health risk associated with surface-water nitrate exposure increased 10-fold from 6 to 60 million persons per year (Figure 1a). The number of populations at potential chronic health risks was more than an order of magnitude larger than those at potential acute risk (Figure 1a,b). Populations at chronic health risk increased 8-fold from 169 to 1361 million persons per year between 1970 and 2010. The relative increase rates of populations at acute and chronic risks (10-fold and 8-fold) were much faster than the nearly 2-fold increase in the global total populations as well as the 1.6-fold increase in global nitrogen loading to surface waters during this period (Figure S1). Moreover, since 1970, the proportions of global populations potentially affected by acute and chronic risks in total world populations increased 5-fold and 4-fold to 1% and 20% in 2010, respectively (Figure 3a).

### 3.2 Continental Populations at Potential Risks Associated with Surface-Water Nitrate Exposure

#### 3.2.1 Continental Populations at Potential Acute Risk

During 1970–2010, world populations potentially affected by acute risk associated with surface-water nitrate exposure were predominantly in Asia, with its contribution increasing from 60% (∼4 million persons per year) to 75% (45 million persons per year; Figure 1a). The second largest contribution to populations at potential acute risk was in Europe in 1970; European populations at potential acute risk increased from 1 to 6 million persons per year during 1970–1990 and gradually decreased to 2 million persons per year in 2010. North and South American populations at potential acute risk showed temporal patterns similar to those in Europe. African populations at potential acute risk showed a continuous increase similar to those in Asia, and their global share increased rapidly from 1% (<0.1 million persons per year) in 1970 to 17% (10 million persons per year) in 2010. Despite an increase, Oceanian contribution (<0.1%) was negligible during 1970–2010.

#### 3.2.2 Continental Populations at Potential Chronic Risk

In 1970, world populations at potential chronic risk associated with surface-water nitrate exposure were mainly in Europe, with its share of up to 61% (Figure 1b). European populations at chronic risk increased 2-fold during 1970–1990 and decreased from 228 million persons per year in 1990 to 129–140 million persons per year during 2000–2010. Asian populations at chronic risk increased rapidly from 40 to 1061 million persons per year during the entire period of 1970–2010, and its share in global populations at chronic risk increased from 24% (second largest) in 1970 to 78% (largest) in 2010. Asia and Europe accounted for more than 80% of global populations at potential chronic risk during 1970–2010, with the rest in North America (5–11%), Africa (3–4%), South America (1–4%), and Oceania (<1%). While North American populations at chronic risks showed a temporal pattern similar to those in Europe, African, South American, and Oceanian populations at chronic risks showed rapid increases similar to those in Asia.

#### 3.2.3 Proportions of Continental Populations at Potential Acute and Chronic Risks

Between 1970 and 2010, the ratios of populations potentially affected by acute risk to those affected by chronic risk showed a large increase in Africa (from 1% to 18%), a modest increase in Europe (from 1% to 2%), and an unchanged value in North America (4%). In contrast, this ratio decreased in Asia, South America, and Oceania (Figure 1c,d).

The proportion of populations potentially affected by chronic risk in total populations was largest in Europe during 1970–2000, with a peak value of 40% in 1990 and values of around 20% in other years (Figure 1e). Temporal changes in the chronic proportions were similar in Europe and North America, with an increase during 1970–1990, a decline during 1990–2000, and stabilization thereafter. In contrast, the chronic proportions increased in Asia, Africa, South America, and Oceania during the entire period of 1970–2010. As a result, in 2010, the largest chronic proportion (25%) was found in Asia.

North America had the largest acute proportion (in its total population) among the continents in 1970 (0.3%), 1990 (1.7%), and 2000 (1.2%; Figure 1e). In other years, Africa and Asia ranked the highest in their proportions of populations at acute risks (in their total populations). This shift in continental dominance is related to their different temporal patterns. The acute proportions in Europe and North America peaked in 1990 and sharply decreased from 1990 onward. South American acute proportion peaked also in 1990, decreased during 1990–2000, and was stable during 2000–2010. Asian acute proportion increased during 1970–2000 and slightly decreased thereafter, so that its decrease was later than those of North America, Europe, and South America. African and Oceanian acute proportions showed increases similar to their chronic proportion patterns, with no decreasing trends yet.

Despite diverse temporal patterns of acute and chronic proportions for different continents, the acute and chronic proportions in 2010 were larger than those in 1970 for all continents except for the acute proportion in South America (Figure 1e).

### 3.3 National Populations at Potential Risks Associated with Surface-Water Nitrate Exposure

#### 3.3.1 National Populations at Potential Acute Risks

**Figure 2:**
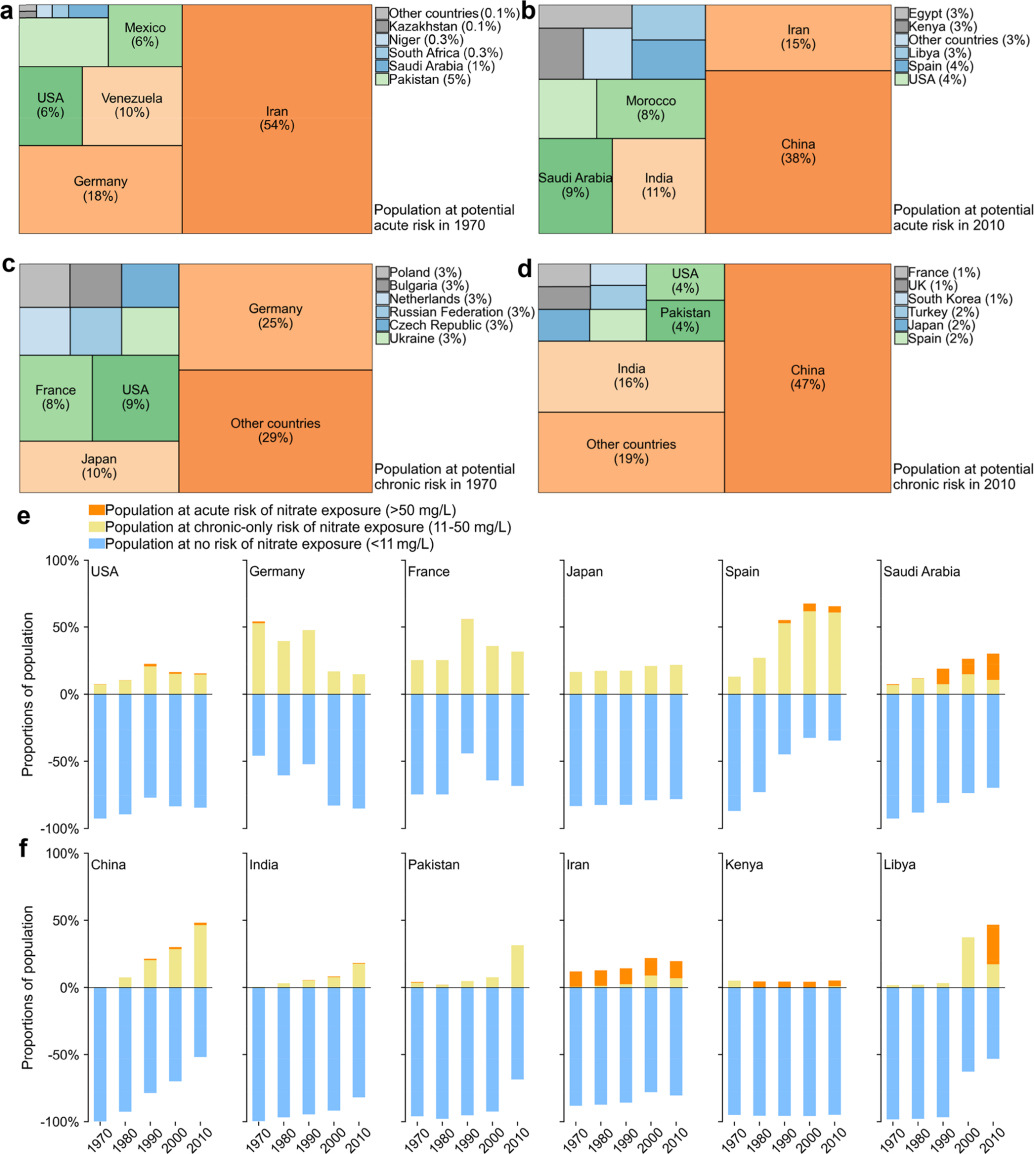
World populations in different countries at potential acute (a, b) and chronic (c, d) risks associated with surface-water nitrate exposure in 1970 (a, c) and 2010 (b, d) and temporal changes in the proportions of populations at potential no risk, chronic-only risk, and acute risk associated with surface-water nitrate exposure in high- (e) and middle-income countries (f). In (a–d), the affected populations of the top-ten countries are shown explicitly by both their areas and colors, and the affected populations from the rest of the countries are summarized as the part of “other countries”. The largest rectangles (representing the countries with the largest affected populations worldwide) are placed in the bottom right and arranged in order of decreasing size up toward the top left; the top three are shown in orange, the fourth–sixth countries are shown in green, the seventh–ninth are shown in blue, and the rest are shown in gray. Exposed populations shown in the part of “other countries” are from 10 (a), 16 (b), 78 (c), and 98 (d) countries in the corresponding figures. The percentages in the rectangle boxes (for the top five countries) or legend labels (for the top sixth–tenth countries and “other countries”) represent the proportions of the country’s affected populations in the global total affected populations by acute or chronic risk in the corresponding year. The countries in (e,f) are selected from the top-ten countries in (a–d). In (e,f) populations at chronic-only risk are populations at chronic risk excluding those at acute risk.

**Figure 3:**
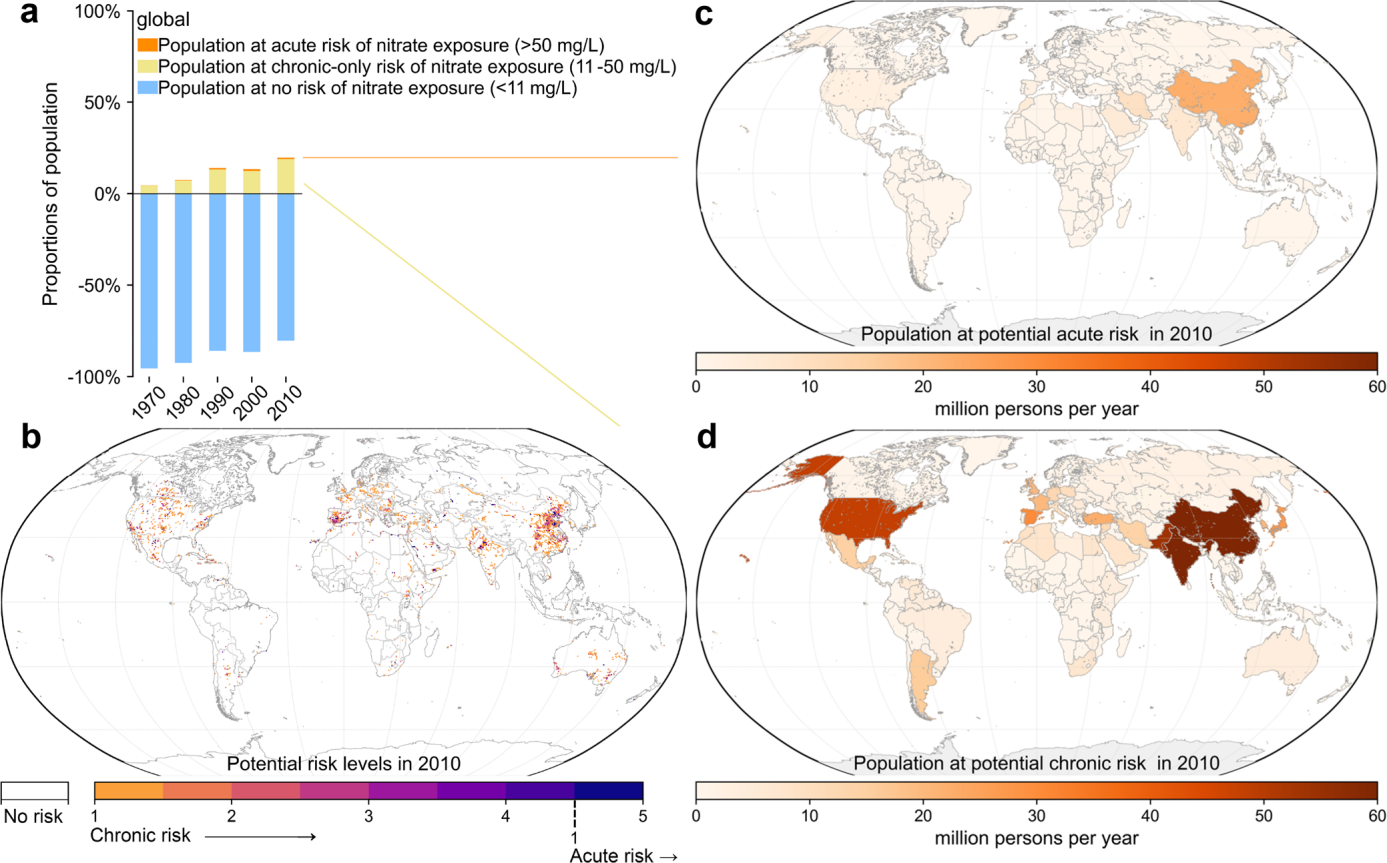
Temporal changes in the proportions of populations at potential no risk, chronic-only risk, and acute risk associated with surface-water nitrate exposure during 1970–2010 (a), distributions of potential risk level (PRL) of surface-water nitrate exposure at a 0.5° × 0.5° resolution (b), and national distributions of populations at acute (c) and chronic (d) risks associated with surface-water nitrate exposure in 2010. In (a), populations at chronic-only risk are populations at chronic risk excluding those at acute risk.

During 1970–2010, world populations potentially affected by acute risks associated with surface-water nitrate exposure were mainly in countries in the northern Hemisphere (Figure 3c). In 1970, Iran (in Asia) contributed to over half of the world’s population that was potentially affected by acute risk associated with surface-water nitrate exposure (Figure 2a). This is related to Iran’s high proportion (>10%) of populations at acute risk in its total populations (Figure 2f). Germany (in Europe) contributed 18%, Venezuela (in South America) contributed 10%, and the rest of the countries together contributed less than 20% (Figure 2a).

Since 1970, the number of countries whose inhabitants were potentially affected by acute risks associated with surface-water nitrate exposure increased 1.3-fold to 26 in 2010 (Figure 2a,b). Moreover, the importance of countries shifted to dominance by four Asian countries: China (38%), Iran (15%), India (11%), and Saudi Arabia (9%). The rest was mainly from African countries, USA, and Spain. This shift is a result of disproportionate changes in national populations,^69^ decreased proportions of populations at acute risk in Germany (Figure 2e), and increased proportions of populations at acute risk in China, India, Saudi Arabia, and African countries (Figure 2f).

#### 3.3.2 National Populations at Potential Chronic Risks

During 1970–2010, countries in the northern Hemisphere accounted for ∼95% of world populations at potential chronic risks associated with surface-water nitrate exposure (Figure 3d). Many more countries were potentially affected by chronic risks than by acute risks (Figures 2 and 3). In 1970, potential chronic health risks affected populations in more than one-fourth of world countries. In 2010, this percentage increased 1.2-fold to more than one-third of world countries.

Moreover, countries affected by potential chronic risks differed from those affected by potential acute risks. In 1970, high-income countries Germany (25%, in Europe), Japan (10%, in Asia), the USA (9%, in North America), and France (8%, in Europe) dominated populations at chronic risks (Figure 2c). The rest was mainly from other European countries (28%), followed by countries in other continents. As a result of the dual increases in total populations^69^ and their proportions of populations at chronic risk in middle-income Asian countries (Figure 2f), China (47%), India (16%), and Pakistan (4%) dominated the world’s population potentially affected by chronic risks in 2010 (Figure 2d). The rest were from high- or upper-middle-income countries in Europe, North America, and Asia. The high-income countries Germany, USA, and France had smaller shares in world populations at potential chronic risk in 2010 than in 1970, due to their reductions in proportions of populations at chronic risk (Figure 2e). The share of Japan in world populations at potential chronic risk also decreased during 1970–2010 because the fraction of Japan’s national population in the global total population decreased^69^ while Japan’s proportion of populations at chronic risk was stable during 1970–2010 (Figure 2e).

### 3.4 Spatial Distributions of Potential Risk Levels of Surface-Water Nitrate Exposure

In the last year covered in this study, 2010, high potential chronic risks were mainly concentrated in eastern and southern Asia, Europe, and central and southern Northern America (Figure 3b). Potential chronic risks also emerged in other continents at locations with dense populations,^69^ water scarcity,^71^ or high nitrogen loading from agricultural activities and wastewater.^72^ Potential acute risks were much more spatially concentrated than were potential chronic risks (Figure 3b). Hotspots of potential acute risks were at locations with both high anthropogenic loading^72^ and water scarcity,^71^ typically in northern coastal China, coastal regions in countries bordering the Mediterranean Sea, Red Sea, and Arabian Gulf, as well as a few inland locations of USA, China, and India. Despite a relatively small areal coverage, hotspots of potential acute risks were densely populated and thus affected considerable populations of 60 million persons globally (Figure 1a).

## 4 Discussion

### 4.1 Comparison of Estimates

Our findings indicate that surface-water nitrate exposure to world populations has expanded and intensified since 1970. These spatial and temporal trends in surface-water exposure have posed increased acute and chronic health risks with a shift in dominance from high-income countries (in Europe and North America) to middle-income countries (in Asia and Africa).

No previous global-scale estimates of surface-water nitrate exposure to world populations are available for comparison. Our estimates suggest that Asia had the largest population at acute risk associated with surface-water nitrate exposure in 2010, followed by Africa, North America, Europe, and other continents. This regional variation is consistent with that of global deaths due to unsafe water sources estimated by a recent study for the year 2019.^4^ However, the death estimates by Wolf et al.^4^ included the overall adverse health outcomes related to unsafe water sources, while this study focused specifically on nitrate overexposure through water.

### 4.2 Surface-Water Nitrate Exposure versus Groundwater and Drinking-Water Nitrate Exposure

This study serves as a first step toward understanding surface-water nitrate exposure to world populations. We estimated that in 2010, 60 million persons were at potential acute health risks and 1361 million persons were at potential chronic health risks associated with surface-water nitrate exposure on the global scale.

Today, surface water and groundwater are equally important as global drinking water supplies.^6^ Groundwaters in shallow, rural domestic wells in agricultural areas are often reported to be contaminated with nitrate,^73−76^ with their nitrate concentrations being even higher than those in surface waters. The high fertilizer use and manure production in historical and contemporary agricultural activities have further contributed to the persisting high groundwater nitrate concentrations in these areas.^77^ Pennino et al.^78^ show that in the USA 95% of nitrate standard violations are in groundwater-sourced drinking water, with the rest in surface-water systems, and that 35% and 65% of people affected by drinking-water nitrate standard violations are served on groundwater and surface-water systems, respectively. If groundwater and surface water are both considered in the total public and private water supplies for nitrate exposure estimation, the estimated waterborne nitrate exposure and affected populations might be larger than the surface-water nitrate exposure estimated in this study. However, overall waterborne nitrate exposure remains highly elusive in different regions and periods. First, there could be an overlap between populations solely affected by surface-water exposure and those affected by total exposure (of surface water and groundwater). Second, in some regions, the proportion of groundwater use can be very limited/variable in the total water supply due to either groundwater scarcity or a preference for surface water. The lack of spatial and temporal data of groundwater importance in drinking water and associated nitrate concentrations adds more challenges to gaining a full understanding. As data of groundwater supply for drinking and nitrate concentrations become increasingly available through monitoring^65^ and modeling efforts,^79,80^ it may be possible to explore and evaluate groundwater nitrate exposure and its synthesis with surface-water nitrate exposure in the future.

Moreover, it is important to recognize that the national and regional differences in technology and capability of drinking water treatment^4^ as well as their changes with time may play a role in drinking-water nitrate exposure to world populations. High-income countries or regions may be more capable of adopting nitrate removal in addition to conventional municipal water treatment and handling the disadvantages of nitrate removal treatment such as high costs, operational complexities, and the disposal of resin, brine, or reject water,^7^ while some low-income countries or regions may not even have any managed drinking water service.^1^ On the other hand, ammonia and nitrification in the distribution systems may increase the drinking-water nitrate concentration.^67^ These may cause possible discrepancies between the estimate of drinking-water nitrate exposure and the estimate of nitrate exposure from surface waters or the public water supply. To improve this drinking-water nitrate estimation, future research that combines associated national and regional monitoring, administrative, and survey data with modeling approaches is warranted.

### 4.3 Implications for (Inter)national Nitrate Standards for Health Risks

WHO and many national agencies recommend 45 or 50 mg/L as the limit for nitrates in drinking water.^7,19−23^ These guidelines assume that exposure of nitrate below this level will not cause significant health problems. They do not consider chronic health risks of various cancers, birth defects, and spontaneous abortions.^24^

Epidemiological studies have increasingly reported various adverse health effects well below the nitrate guidelines^24^ (Table S2). In this study, we consider nitrate overexposure as a cofactor that potentially increases these adverse health effects. Despite uncertainties related to the reported thresholds of various chronic effects, our quantitative assessment showed that chronic risks associated with surface-water nitrate exposure would affect 20-fold more populations than acute risks on a global scale. Considering the role of nitrate overexposure as a potential cofactor in the exposome, the standards of maximum nitrate contamination may need to be lowered.

If such a lower nitrate standard is adopted to better protect public health, world populations at potential chronic risks might be expected to decline in the coming future. This adjustment of standards, however, requires support from long-term epidemiological research capable of tracking chronic risks to populations with a (changing) historical nitrate exposure. Particular attention should be given to the trajectory of the subpopulation most susceptible to adverse health effects from nitrate overexposure.^14,33,34,36−38,40,41,81,82^

Moreover, today, in countries with well-regulated water distribution networks, nitrate in surface waters is probably treated to meet the (inter)national standard (45 or 50 mg/L as mentioned), which is not protective against chronic effects. If a lower nitrate standard such as 11 mg/L is adopted and enforced to protect health against both acute and chronic risks, merely reducing anthropogenic inputs to surface waters will be insufficient to achieve the goal, considering the input of historically accumulated nitrate from groundwater with long residence times.^77^ Current nitrate removal technologies can achieve effluent nitrate concentrations as low as 13 mg/L,^7^ which may still not be sufficiently low. Therefore, most countries and drinking water providers will need to intensify investments in advanced treatment technologies to enhance nitrate removal efficiency, especially if they do not necessarily control their upstream conditions.

In some high-risk regions identified in this study, a sufficient decline of surface-water nitrate concentrations might not be expected in the short run, possibly due to persisting anthropogenic nitrogen loading, large water stress, and/or limited water treatment capabilities.^72,83,84^ For inhabitants in these regions, reduction of surface-water consumption, a shift to bottled purified water, and the use of water purifiers at home for drinking and cooking may be effective protective measures. For the particularly susceptible subpopulations of infants, it is recommended that formulas be prepared with purified bottled water. Besides, some studies show that potential health risks related to nitrate exposure tend to be lower for those who consume high amounts of fiber, fruits, and vegetables, which contain antioxidants, polyphenols, and vitamins as protective agents.^41,85^ Inhabitants at potential chronic and/or acute risks related to nitrate exposure may therefore consider a dietary transformation to more fiber, fruits, and vegetables. However, some other studies suggest that dietary intake of leafy vegetables, which are reported to be nitrate-accumulating, is likely another significant source of nitrate exposure,^8,86,87^ and warrants careful consideration alongside nitrate exposure from water intake.

### 4.4 Sources of Surface-Water Nitrate Exposure

Nitrate in surface waters can originate from both diffuse and point nitrogen sources on the land.^61,88^ Globally, diffuse sources are dominant, with the largest contribution from agriculture due to the excessive use of fertilizer, expansion of agricultural land, and massive manure from livestock.^61,88^ However, point sources of nitrate from wastewater discharge have increased rapidly since the 1970s due to the rapid increase in population, urbanization, and industrialization.^88^ Moreover, nitrates can also be generated within surface waters through transformations from other reactive nitrogen forms.^55^ Anthropogenic diffuse and point sources have driven increased organic nitrogen and ammonium loading to surface waters.^88^ Their in-stream transformations and associated in-stream production of nitrate have increased with the result that most nitrate is now formed within freshwater bodies.^56^ These anthropogenic impacts on raising surface-water nitrate concentrations, in turn, pose increased health risks to humans.

To mitigate adverse health effects associated with nitrate exposure from surface waters, anthropogenic nitrogen inputs to natural environments should be drastically reduced. This requires long-term efforts of improvements in wastewater treatment, agricultural and environmental management practices, and nutrient recycling from waste,^77,89,90^ particularly in the regions and countries with increasing populations at potential health risks associated with surface-water nitrate exposure. Besides, this needs to be combined with improvement in drinking-water treatment technology, considering the time lag between source control and water quality improvement.^77^

## Supporting Information Available

Global population, gross production value of agriculture, wastewater nitrogen discharge, and total nitrogen loading to surface waters during 1970–2010, spatial distributions of simulated nitrate concentrations in global surface waters between 1970 and 2010, comparison of simulated and observed nitrate concentrations per site per year at surface-water monitor sites in major river basins worldwide since the 1950s, information and sources of the observation data used for validation of surface-water nitrate concentrations worldwide, and summary of potential health risks associated with high waterborne nitrate exposure and their corresponding threshold nitrate concentrations above which elevated risks were reported in the literature (PDF)
