# Supplementary material for: Surface-Water Nitrate Exposure to World Populations Has Expanded and Intensified during 1970–2010
Source: Environ Sci Technol. 2023 Nov 21;57(48):19395–406. doi: 10.1021/acs.est.3c06150 (PMC10702521; doi:10.1021/acs.est.3c06150)
Supplement: Supplementary file 1 — es3c06150_si_001.pdf [file es3c06150_si_001.pdf]

Supporting Information for

Surface-water nitrate exposure to world populations has expanded and intensified during 1970-2010

Junjie Wang[1], Xiaochen Liu[1]*, Arthur H.W. Beusen[1,2], Jack J. Middelburg[1]*

[1] Department of Earth Sciences, Utrecht University, Princetonlaan 8a, Utrecht, The Netherlands, 3584CB

[2] PBL Netherlands Environmental Assessment Agency, P.O. Box 30314, The Hague, The Netherlands, 2500GH

*Corresponding author: Junjie Wang (j.wang3@uu.nl), Xiaochen Liu (x.liu@uu.nl)

Supporting Information includes 8 pages, 3 figures, and 2 tables.

Contents:

Supporting Figures

Figure S1. Global population, gross production value of agriculture, wastewater nitrogen discharge, and total nitrogen loading to surface waters during 1970-2010.

Figure S2. Spatial distributions of nitrate concentrations in global surface waters in 1970 and 2010 simulated by IMAGE-DGNM.

Figure S3. Comparison of simulated and observed nitrate concentrations per site per year at surface-water monitor sites in major river basins worldwide since the 1950s.

Supporting Tables

Table S1. Information and sources of the observation data used for validation of surface-water nitrate concentrations per site per year in a series of major river basins worldwide.

Table S2. Summary of potential health risks associated with high waterborne nitrate exposure and their corresponding threshold nitrate concentrations above which elevated risks were reported in literature.

References

Figure S1

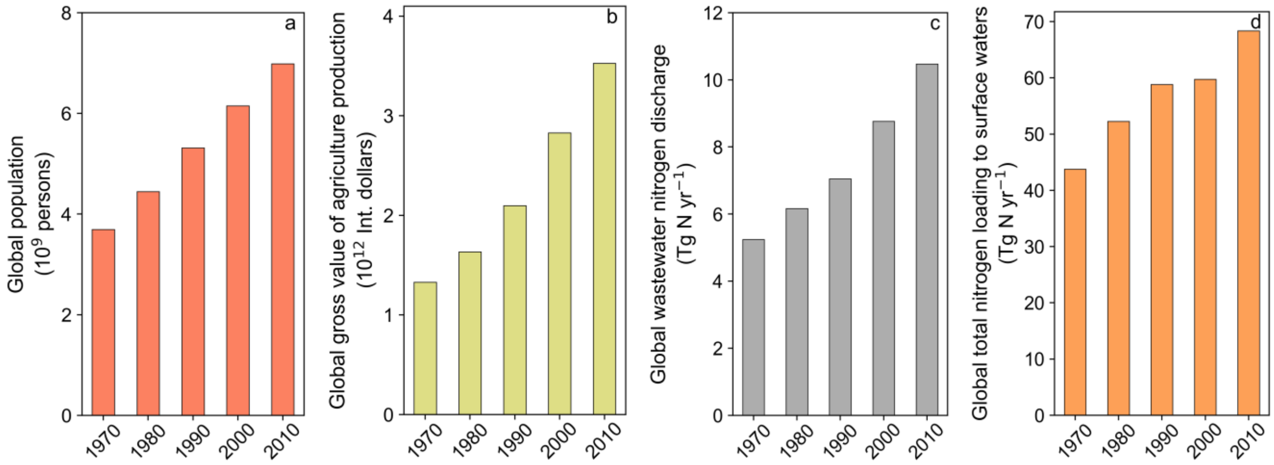

Figure S1. Global population (a), gross value of agriculture production (b), wastewater nitrogen discharge to surface waters (c), and global total nitrogen loading to surface waters (d) during 1970-2010. Data on population and gross value of agriculture production were from FAO (2021). Data on wastewater nitrogen discharge were from van Puijenbroek et al. (2019). Data on global total nitrogen loading to surface waters were from Beusen et al. (2022).

Figure S2

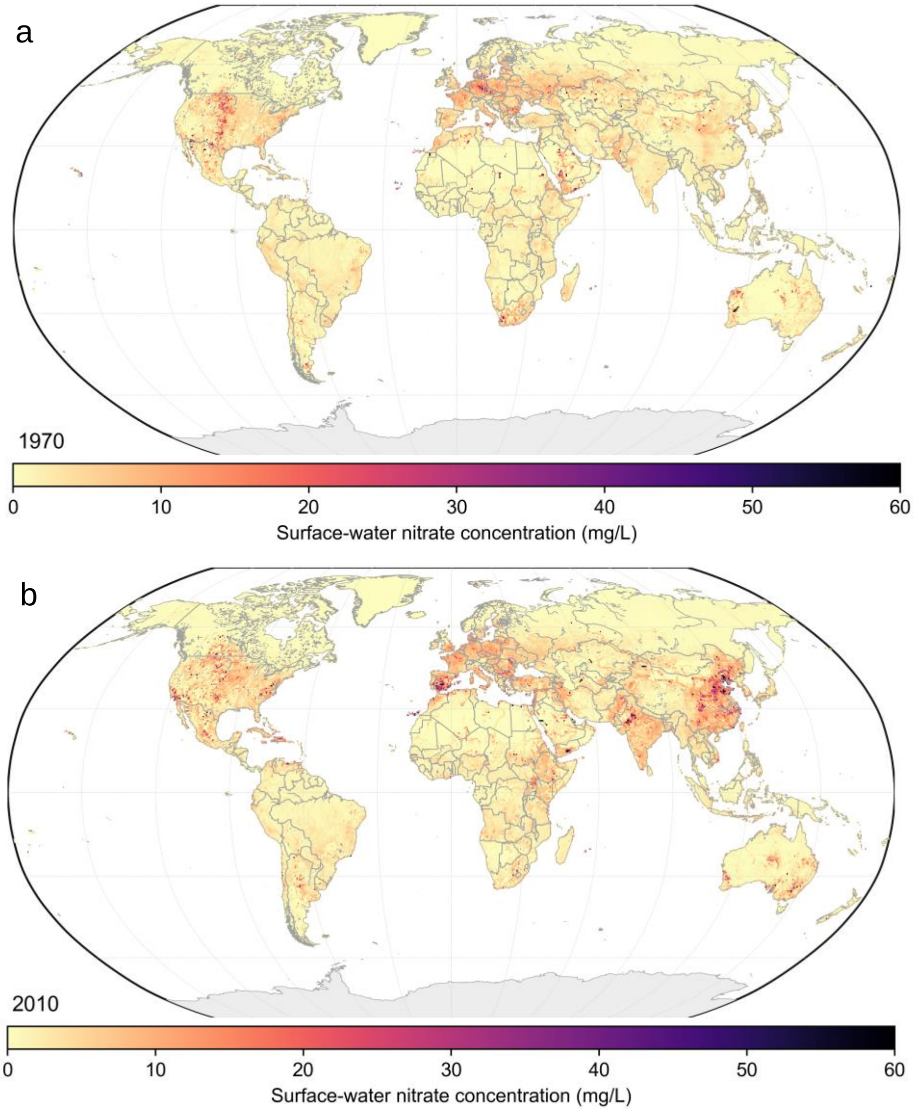

Figure S2. Spatial distributions of nitrate concentrations in global surface waters in 1970 and 2010 simulated by IMAGE-DGNM with a 0.5°×0.5° resolution. Data were from Wang et al. (2022).

Figure S3

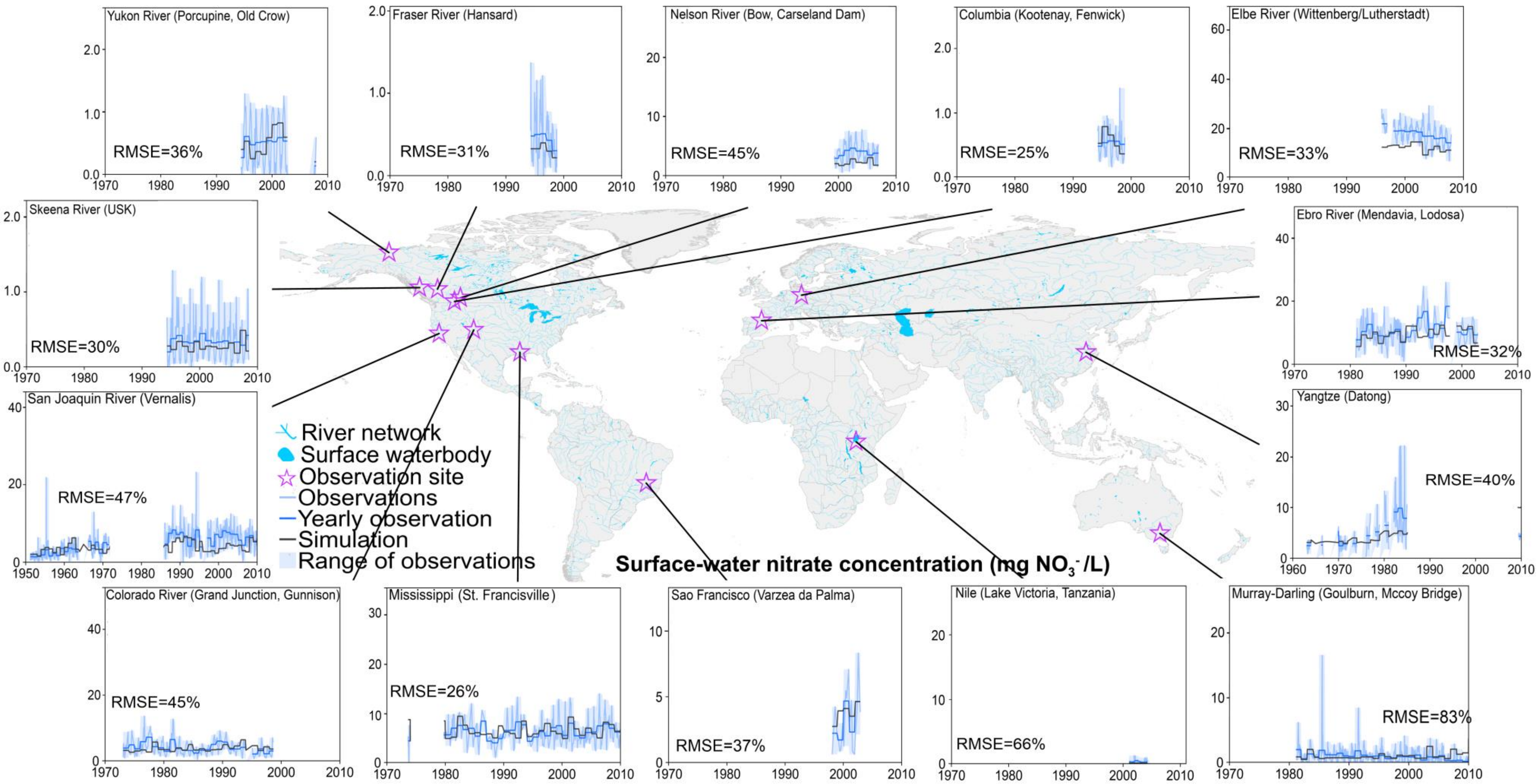

Figure S3. Validation of simulated surface-water nitrate concentrations against observations per site per year at monitor sites in major river basins worldwide since the 1950s. The sites and river basins represent a range in climate, geology, hydrology, and human activities (e.g., land use, agriculture, industry, population). Observations for years with at least four months of observations were used for representativity concerns. The Root Mean Squared Error (RMSE) values of the simulated historical surface-water nitrate concentrations per year and the corresponding yearly average of observations for each site are shown in each validation figure. Information and sources of the observation data are in Table S1.

Table S1. Information and sources of the observation data used for validation of surface-water nitrate concentrations per site per year in a series of major river basins worldwide.

River basin	Site name	Covering period	Data source
Mississippi	St. Francisville	1973-2010	USGS (USGS, 2022)
Nile	Lake Victoria, Tanzania	2001-2004	GEMStat (UNEP, 2018)
Yangtze	Datong	1963-1984, 2009	(Shen et al., 2003; Wu et al., 2023)
Nelson	Bow River, Carseland Dam	1999-2006	GLORICH (Hartmann et al., 2019)
Murray-Darling	Goulburn River, Mccoy Bridge	1981-2010	GLORICH (Hartmann et al., 2019)
Yukon	Porcupine, upstream Old Crow	1994-2007	GLORICH (Hartmann et al., 2019)
Columbia	Kootenay River, Fenwick	1994-1998	GLORICH (Hartmann et al., 2019)
Sao Francisco	Varzea da Palma	1998-2002	GLORICH (Hartmann et al., 2019)
Colorado	Gunnison River, Grand Junction	1973-1998	GLORICH (Hartmann et al., 2019)
Fraser	Hansard	1994-1998	GLORICH (Hartmann et al., 2019)
Elbe	Wittenberg/Lutherstadt	1996-2007	GLORICH (Hartmann et al., 2019)
San Joaquin	Vernalis	1951-2010	USGS (USGS, 2022)
Ebro	Mendavia, Lodosa	1981-2002	GLORICH (Hartmann et al., 2019)
Skeena	USK	1994-2008	GLORICH (Hartmann et al., 2019)

Note: The sites and river basins in Table S1 match their distributions in Figure S3.

Table S2. Summary of potential health risks associated with high waterborne nitrate exposure and their corresponding threshold nitrate concentrations above which elevated risks were reported in the literature.

Adverse health effects	Threshold nitrate concentration for associated risk (mg/L as NO_3^- ion)	Health risk type	Sources
**methemoglobinemia** (primarily infants < 6 months, but also older children, pregnant women and their fetal, individuals with diseases such as gastrointestinal infection, genetic glucose-6-phosphate dehydrogenase deficiency, or reduced gastric acidity, and general adults)	50	evident acute risk	(Comly, 1945; Wolfe and Patz, 2002; Sadeq et al., 2008; WHO, 2022)
**cancers of the digestive tract, e.g., stomach, esophagus, colon and rectum**	3.9-50	strongly potential chronic risk as a co-factor	(Gastric, 1987; Morales-Suarez-Varela et al., 1995; Weyer et al., 2001; Gulis et al., 2002; De Roos et al., 2003; McElroy et al., 2008; Espejo-Herrera et al., 2016; Fathmawati et al., 2017; Taneja et al., 2017; Schullehner et al., 2018)
**cancers of the genitourinary system, e.g., bladder, ovarian, and prostate**	10.9-50	strongly potential chronic risk as a co-factor	(Morales-Suarez-Varela et al., 1995; Weyer et al., 2001; Donat-Vargas et al., 2023; Schmidt, 2023)
**thyroid hypertrophy and cancer**	22.1-50	potential chronic risk as a co-factor	(van Maanen et al., 1994; Zaki et al., 2004; Ward et al., 2010; WHO, 2022)
**non-Hodgkin's lymphoma (NHL)**	22.1-50	potential chronic risk as a co-factor	(Ward et al., 1996; Gulis et al., 2002)
**insulin-dependent diabetes mellitus**	3.4-25	potential chronic risk as a co-factor	(Kostraba et al., 1992; Parslow et al., 1997; van Maanen et al., 2000)
**spontaneous abortions, intrauterine growth restriction, and fetal death** (pregnant women), **brain tumors, central nervous system birth defects, infant death, mutagenicity, teratogenicity, and other birth defects** (with maternal exposure or childhood exposure)	3.5-50	potential chronic risk as a co-factor	(Scragg et al., 1982; Dorsch et al., 1984; Arbuckle et al., 1988; van Maanen et al., 1996; Bukowski et al., 2001; Croen et al., 2001; Brender et al., 2004; Mueller et al., 2004; Sadeq et al., 2008; Stayner et al., 2017; Ebdrup et al., 2022)

References

Arbuckle, T.E., Sherman, G.J., Corey, P.N., Walters, D., Lo, B., 1988. Water nitrates and cns birth defects: A population-based case-control study. Archives of Environmental Health: An International Journal 43, 162-167.

Beusen, A.H.W., Doelman, J.C., Van Beek, L.P.H., Van Puijenbroek, P.J.T.M., Mogollón, J.M., Van Grinsven, H.J.M., Stehfest, E., Van Vuuren, D.P., Bouwman, A.F., 2022. Exploring river nitrogen and phosphorus loading and export to global coastal waters in the Shared Socio-economic pathways. Glob. Environ. Chang. 72, 102426.

Brender, J.D., Olive, J.M., Felkner, M., Suarez, L., Marckwardt, W., Hendricks, K.A., 2004. Dietary nitrites and nitrates, nitrosatable drugs, and neural tube defects. Epidemiology 15.

Bukowski, J., Somers, G., Bryanton, J., 2001. Agricultural contamination of groundwater as a possible risk factor for growth restriction or prematurity. J. Occup. Environ. Med. 43, 377-383.

Comly, H.H., 1945. Cyanosis in infants caused by nitrates in well water. Journal of the American Medical Association 129, 112-116.

Croen, L.A., Todoroff, K., Shaw, G.M., 2001. Maternal exposure to nitrate from drinking water and diet and risk for neural tube defects. Am. J. Epidemiol. 153, 325-331.

De Roos, A.J., Ward, M.H., Lynch, C.F., Cantor, K.P., 2003. Nitrate in public water supplies and the risk of colon and rectum cancers. Epidemiology 14.

Donat-Vargas, C., Kogevinas, M., Castaño-Vinyals, G., Pérez-Gómez, B., Llorca, J., Vanaclocha-Espí, M., Fernandez-Tardon, G., Costas, L., Aragonés, N., Gómez-Acebo, I., Moreno, V., Pollan, M., Villanueva Cristina, M., 2023. Long-Term Exposure to Nitrate and Trihalomethanes in Drinking Water and Prostate Cancer: A Multicase–Control Study in Spain (MCC-Spain). Environ. Health Perspect. 131, 037004.

Dorsch, M.M., Scragg, R.K.R., McMichael, A.J., Baghurst, P.A., Dyer, K.F., 1984. Congenital malformations and maternal drinking water supply in rural south australia: A case-control study. Am. J. Epidemiol. 119, 473-486.

Ebdrup, N.H., Schullehner, J., Knudsen, U.B., Liew, Z., Thomsen, A.M.L., Lyngsø, J., Bay, B., Arendt, L.H., Clemmensen, P.J., Sigsgaard, T., Hansen, B., Ramlau-Hansen, C.H., 2022. Drinking water nitrate and risk of pregnancy loss: a nationwide cohort study. Environmental Health 21, 87.

Espejo-Herrera, N., Gràcia-Lavedan, E., Boldo, E., Aragonés, N., Pérez-Gómez, B., Pollán, M., Molina, A.J., Fernández, T., Martín, V., La Vecchia, C., Bosetti, C., Tavani, A., Polesel, J., Serraino, D., Gómez Acebo, I., Altzibar, J.M., Ardanaz, E., Burgui, R., Pisa, F., Fernández-Tardón, G., Tardón, A., Peiró, R., Navarro, C., Castaño-Vinyals, G., Moreno, V., Righi, E., Aggazzotti, G., Basagaña, X., Nieuwenhuijsen, M., Kogevinas, M., Villanueva, C.M., 2016. Colorectal cancer risk and nitrate exposure through drinking water and diet. Int. J. Cancer 139, 334-346.

FAO 2021. FAOSTAT database collections. Accessed on April 12, 2022. Rome. Food and Agriculture Organization of the United Nations. http://www.fao.org/faostat/en/#data.

Fathmawati, Fachiroh, J., Gravitiani, E., Sarto, Husodo, A.H., 2017. Nitrate in drinking water and risk of colorectal cancer in Yogyakarta, Indonesia. J. Toxicol. Environ. Health, Part A 80, 120-128.

Gastric, F.D., 1987. Gastric cancer, diet, and nitrate exposure. British Medical Journal (Clinical research ed.) 294, 528-529.

Gulis, G., Czompolyova, M., Cerhan, J.R., 2002. An ecologic study of nitrate in municipal drinking water and cancer incidence in Trnava District, Slovakia. Environ. Res. 88, 182-187.

Hartmann, J., Lauerwald, R., Moosdorf, N., 2019. GLORICH - Global river chemistry database. PANGAEA.

Kostraba, J.N., Gay, E.C., Rewers, M., Hamman, R.F., 1992. Nitrate levels in community drinking waters and risk of iddm: An ecological analysis. Diabetes Care 15, 1505-1508.

McElroy, J.A., Trentham-Dietz, A., Gangnon, R.E., Hampton, J.M., Bersch, A.J., Kanarek, M.S., Newcomb, P.A., 2008. Nitrogen-nitrate exposure from drinking water and colorectal cancer risk for rural women in Wisconsin, USA. J. Water Health 6, 399-409.

Morales-Suarez-Varela, M.M., Llopis-Gonzalez, A., Tejerizo-Perez, M.L., 1995. Impact of nitrates in drinking water on cancer mortality in Valencia, Spain. Eur. J. Epidemiol. 11, 15-21.

Mueller, B.A., Nielsen, S.S., Preston-Martin, S., Holly, E.A., Cordier, S., Filippini, G., Peris-Bonet, R., Choi, N.W., 2004. Household water source and the risk of childhood brain tumours: results of the SEARCH International Brain Tumor Study. Int. J. Epidemiol. 33, 1209-1216.

Parslow, R.C., McKinney, P.A., Law, G.R., Staines, A., Williams, R., Bodansky, H.J., 1997. Incidence of childhood diabetes mellitus in Yorkshire, northern England, is associated with nitrate in drinking water: an ecological analysis. Diabetologia 40, 550-556.

Sadeq, M., Moe, C.L., Attarassi, B., Cherkaoui, I., ElAouad, R., Idrissi, L., 2008. Drinking water nitrate and prevalence of methemoglobinemia among infants and children aged 1–7 years in Moroccan areas. Int. J. Hyg. Environ. Health 211, 546-554.

Schmidt, S., 2023. Nitrates and Prostate Cancer: Long-Term Drinking Water Exposures Associated with Risk of Tumors. Environ. Health Perspect. 131, 054003.

Schullehner, J., Hansen, B., Thygesen, M., Pedersen, C.B., Sigsgaard, T., 2018. Nitrate in drinking water and colorectal cancer risk: A nationwide population-based cohort study. Int. J. Cancer 143, 73-79.

Scragg, R.K.R., McMichael, A.J., Baghurst, P.A., Dorsch, M.M., 1982. Birth defects and household water supply Epidemiological studies in the Mount Gambier region of South Australia. Med. J. Aust. 2, 577-579.

Shen, Z., Liu, Q., Zhang, S., 2003. Distribution, variation and removal patterns of inorganic nitrogen in the Changjiang River. Oceanologia et Limnologia Sinica 34(4), 355-363 (in Chinese).

Stayner, L.T., Almberg, K., Jones, R., Graber, J., Pedersen, M., Turyk, M., 2017. Atrazine and nitrate in drinking water and the risk of preterm delivery and low birth weight in four Midwestern states. Environ. Res. 152, 294-303.

Taneja, P., Labhasetwar, P., Nagarnaik, P., Ensink, J.H.J., 2017. The risk of cancer as a result of elevated levels of nitrate in drinking water and vegetables in Central India. J. Water Health 15, 602-614.

UNEP, U.N.E.P. 2018. GEMStat database of the Global Environment Monitoring System for Freshwater (GEMS/Water) Programme. (International Centre for Water Resources and Global Change, Koblenz.).

USGS 2022. United States Geological Survey: Water Quality Samples for the Nation. (United States Geological Survey).

van Maanen, J.M., Albering, H.J., de Kok, T.M., van Breda, S.G., Curfs, D.M., Vermeer, I.T., Ambergen, A.W., Wolffenbuttel, B.H., Kleinjans, J.C., Reeser, H.M., 2000. Does the risk of childhood diabetes mellitus require revision of the guideline values for nitrate in drinking water? Environ. Health Perspect. 108, 457-461.

van Maanen, J.M., Welle, I.J., Hageman, G., Dallinga, J.W., Mertens, P.L., Kleinjans, J.C., 1996. Nitrate contamination of drinking water: relationship with HPRT variant frequency in lymphocyte DNA and urinary excretion of N-nitrosamines. Environ. Health Perspect. 104, 522-528.

van Maanen, J.M.S., van Dijk, A., Mulder, K., de Baets, M.H., Menheere, P.C.A., van der Heide, D., Mertens, P.L.J.M., Kleinjans, J.C.S., 1994. Consumption of drinking water with high nitrate levels causes hypertrophy of the thyroid. Toxicol. Lett. 72, 365-374.

van Puijenbroek, P., Beusen, A.H.W., Bouwman, A.F., 2019. Global nitrogen and phosphorus in urban waste water based on the Shared Socio-economic pathways. J. Environ. Manag. 231, 446-456.

Wang, J., Bouwman, A.F., Vilmin, L., Mogollón, J.M., Beusen, A.H.W., van Hoek, W.J., Liu, X., Pika, P.A., Middelburg, J.J., 2022. Accelerated nitrogen cycle in global river basins in the Anthropocene. AGU Fall Meeting 2022.

Ward, M.H., Kilfoy, B.A., Weyer, P.J., Anderson, K.E., Folsom, A.R., Cerhan, J.R., 2010. Nitrate intake and the risk of thyroid cancer and thyroid disease. Epidemiology 21.

Ward, M.H., Mark, S.D., Cantor, K.P., Weisenburger, D.D., Correa-Villaseñor, A., Zahm, S.H., 1996. Drinking water nitrate and the risk of non-Hodgkin's lymphoma. Epidemiology 7.

Weyer, P.J., Cerhan, J.R., Kross, B.C., Hallberg, G.R., Kantamneni, J., Breuer, G., Jones, M.P., Zheng, W., Lynch, C.F., 2001. Municipal drinking water nitrate level and cancer risk in older women: The Iowa women's health study. Epidemiology 12, 327-338.

WHO, W.H.O. 2022. Guidelines for drinking-water quality: Fourth edition incorporating the first and second addenda (Geneva, World Health Organization.), p. 631.

Wolfe, A.H., Patz, J.A., 2002. Reactive nitrogen and human health: Acute and long-term implications. AMBIO: A Journal of the Human Environment 31, 120-125.

Wu, W., Wang, J., Wang, H., Liu, J., Yao, Q., Yu, Z., Ran, X., 2023. Trends in nutrients in the Changjiang River. Sci. Total Environ. 872, 162268.

Zaki, A., Ait Chaoui, A., Talibi, A., Derouiche, A.F., Aboussaouira, T., Zarrouck, K., Chait, A., Himmi, T., 2004. Impact of nitrate intake in drinking water on the thyroid gland activity in male rat. Toxicol. Lett. 147, 27-33.
